# A Rare Case of Dyggve-Melchior-Clausen Syndrome: A Case Report

**DOI:** 10.7759/cureus.69495

**Published:** 2024-09-16

**Authors:** Sanjay Chavan, Shiji Chalipat, Sarnya Verma, Gaurav Kumar, Shailaja Mane

**Affiliations:** 1 Pediatrics, Dr. D. Y. Patil Medical College, Hospital and Research Centre, Dr. D. Y. Patil Vidyapeeth (Deemed to be University), Pune, IND

**Keywords:** dyggve-melchior-clausen syndrome, dym gene, morquio syndrome, rare genetic disorder, skeletal abnormalities

## Abstract

Dyggve-Melchior-Clausen (DMC) disease, also known as DMC syndrome, is a rare, progressive genetic disorder that is characterized by skeletal and intellectual abnormalities. The case report involves a four-year-old male child presenting with marked short stature, intellectual disability, coarse facies, and microcephaly. Initial investigations, including blood tests and radiological evaluations, prompted further genetic testing via whole-exome sequencing. This identified a homozygous mutation in the Dymeclin (*DYM*) gene, implicating DMC disease. The condition usually poses diagnostic challenges due to overlapping clinical features with Morquio syndrome. This case highlights the importance of a comprehensive diagnostic approach and genetic testing in elucidating the underlying genetic etiology of complex presentations in pediatric patients.

## Introduction

Dyggve-Melchior-Clausen disease (DMC), also known as DMC syndrome, is a rare genetic disorder characterized by progressive spondyloepimetaphyseal dysplasia (SEMD), coarse facies, and intellectual disability [1]. Inherited in an autosomal recessive manner, mutations in the *DYM* gene impair the activity of the Dymeclin protein [2]. Common manifestations include a barrel-shaped chest, short trunk, partial hip dislocation, genu valgum or varum, and scoliosis [3]. Morquio syndrome (mucopolysaccharidosis IV A and B) closely resembles DMC, and due to overlapping features, a high clinical suspicion is required to differentiate between the two conditions. Accurate diagnosis often necessitates a comprehensive clinical, biochemical, radiological, and genetic evaluation. This report presents a case of a child with genetically confirmed DMC syndrome.

## Case presentation

**Figure 1:**
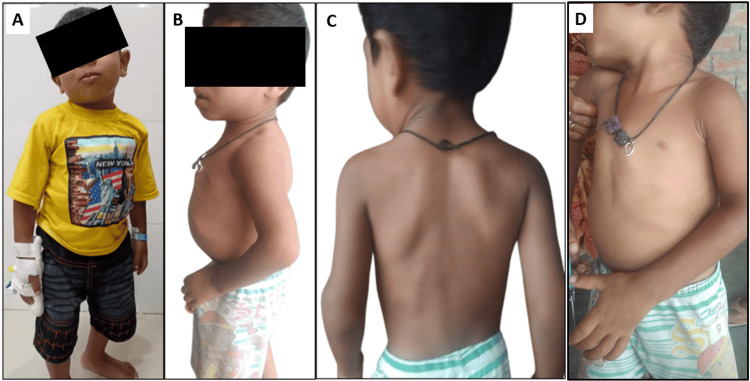
Clinical picture of the patient (A) The front view shows coarse facies; (B) the side view shows abnormal curvature of the spine and flaring of the lower ribs; (C) shows scoliosis; and (D) shows a widening of the wrist

A four-year-old male child, born of a third-degree consanguineous marriage, presented with poor growth and global developmental delay with a birth weight of 2.4 kg and uneventful birth and perinatal histories. Family history was insignificant. Developmental history revealed delayed attainment of all milestones with predominant cognitive delay. On examination, he had coarse facies, microcephaly, café-au-lait macules, and bony deformities with flaring of lower ribs, short hands and feet, widening of wrist and ankle, and prominent thoracic scoliosis (Figure 1). Anthropometric measurements were consistent with findings of severe short stature (with a height of 80 cm, <3 SD), microcephaly (head circumference of 44.5 cm, <3 SD), and underweight for age and sex (a weight of 10.7 kg, <3 SD). No focal neurological deficits were found on neurological examination. The child had no cataract or corneal clouding and no hepatosplenomegaly. Routine blood investigations, including thyroid function tests, yielded normal results (Table 1). Urinary glycosaminoglycan (GAG) analysis was done to screen mucopolysaccharidosis, which yielded negative results. Growth hormone levels were within normal range (28 ng/mL). The karyotyping report was also normal.

**Table 1:** Routine blood investigations WBC: white blood cells; T3: triiodothyronine; T4: tetraiodothyronine; TSH: thyroid-stimulating hormone

Parameter	Value	Reference Range
Hemoglobin	12 g/dL	12.0-14.5 g/dL
WBC	9800/µL	4000-10800/µL
Neutrophils	29%	35%–80%
Lymphocytes	47%	40%–60%
Platelets	443000/µL	400000-530000/µL
Sodium	135 mmol/L	135-145 mmol/L
Potassium	4.3 mmol/L	3.5-5.1 mmol/L
Chloride	104 mmol/L	98-107 mmol/L
Calcium	9.6 mg/dL	8.6-10.2 mg/dL
Phosphate	2.8 mg/dL	2.6-4.7 mg/dL
Alkaline phosphatase	106 U/L	40-129 U/L
T3	1.12 ng/mL	1.13-1.89 ng/mL
T4	6.28 µg/dL	6.0-14.7 µg/dL
TSH	1.47 micro IU/mL	0.7-6.0 micro IU/mL

**Figure 2:**
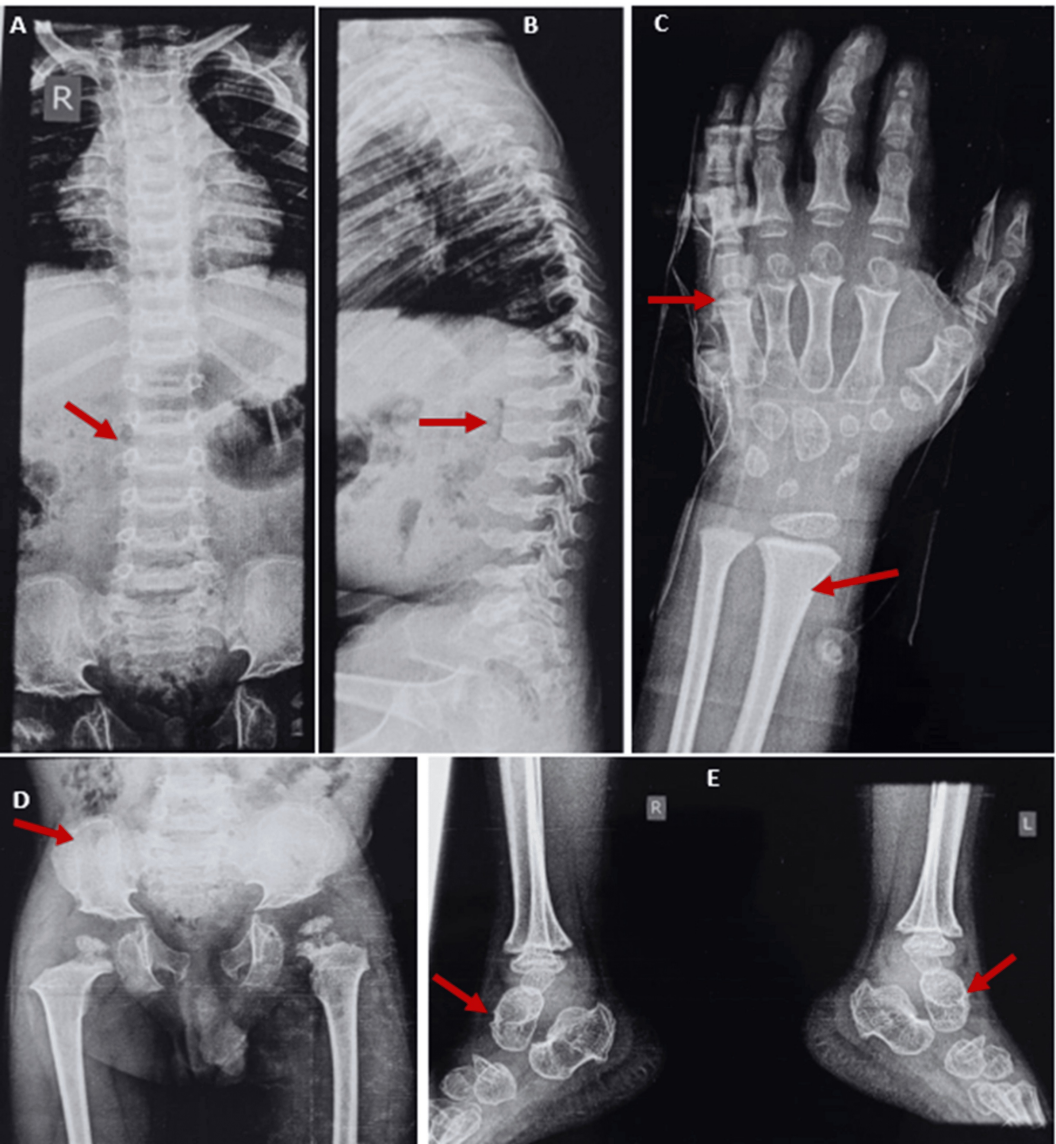
X-ray images (A,B) X-ray spine anteroposterior and lateral views showing scoliosis, anterior beaking, and double-humped thoracic and lumbar vertebral bodies. (C) X-ray hand and wrist anteroposterior view showing short and thick metacarpals, bullet-shaped phalanges, and dysplastic radial and ulnar epiphyses. (D) X-ray of the pelvis showing the non-visualization of the lower sacral vertebrae, an abnormal pelvic inlet, widening of the pubic symphysis, along with a lacy pattern of the iliac crest, a short femoral neck, and irregular femoral epiphysis and metaphysis. (E) X-ray of the bilateral foot and ankle joint revealed a congenital vertical talus

A radiographic skeletal survey was done. The spine X-ray (Figures 2A, 2B) revealed scoliosis, anterior beaking of multiple vertebral bodies, and a double-humped appearance of the thoracic and lumbar vertebral bodies. Additionally, the lower sacral vertebrae were not visualized. An X-ray of the left hand and wrist (Figure 2C) demonstrated irregular carpals and metacarpals, bullet-shaped phalanges, and dysplastic radial and ulnar epiphyses. The pelvis X-ray (Figure 2D) also showed a widening of the pubic symphysis and abnormal pelvic inlet. Non-visualization of the sacral vertebrae raised suspicion of sacral agenesis. The thinning and lace-like appearance of the iliac crest, a feature typically seen in DMC syndrome, was noted. The presence of a short femoral neck, irregular femoral epiphysis and metaphysis, flat acetabular roof, and lateral displacement of femoral heads were suggestive of epiphyseal or metaphysical dysplasia. An X-ray of the ankle (Figure 2E) showed a congenital vertical talus. MRI of the brain and abdominal ultrasonography revealed no abnormalities. Based on the radiological findings, we considered the diagnosis of SEMD (e.g., DMC syndrome, spondyloepiphyseal dysplasia tarda, and Smith-McCort dysplasia) and Morquio syndrome.

**Figure 3:**
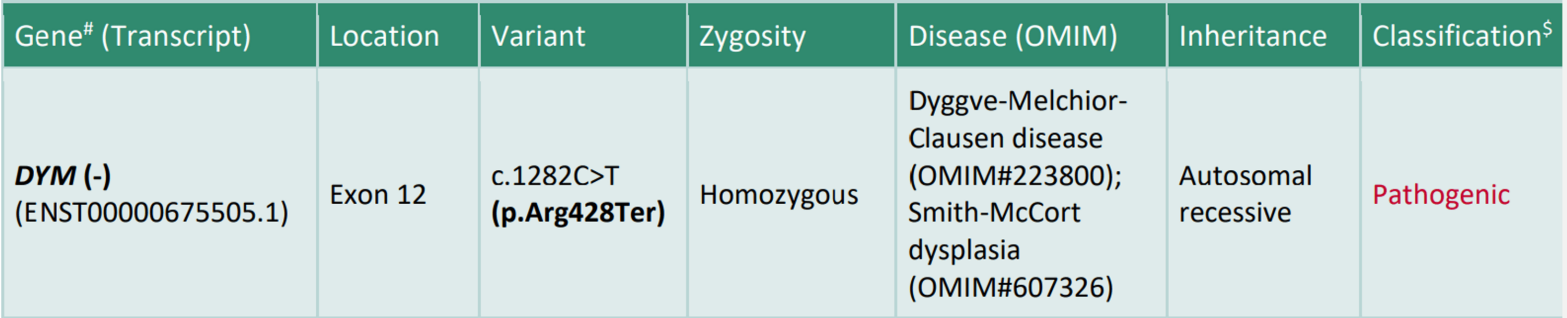
Whole-exome sequencing report

The clinical presentation, distinctive skeletal phenotype, absence of corneal clouding, presence of intellectual disability, and lack of urinary glycosaminoglycans (GAGs) pointed towards an alternative diagnosis. Whole-exome sequencing was performed that confirmed DMC syndrome by identifying a pathogenic homozygous mutation in the *DYM* gene located in exon 12 (Figure 3). The parents were subsequently counseled on Sanger sequencing and prenatal genetic testing for future pregnancies.

## Discussion

DMC syndrome is a rare, autosomal recessive, progressive SEMD with distinctive intellectual disabilities and skeletal deformities that impede normal growth and development. The hallmark structural abnormalities are observed within the spine and pelvis, including abnormalities in the vertebrae and abnormal development of the epiphyses [4]. This can result in spinal curvature abnormalities such as kyphosis and scoliosis, as well as premature degenerative changes within the intervertebral discs [5]. The severity and specific manifestations of SEMD can vary widely among affected individuals, with some experiencing mild skeletal abnormalities and others facing more profound functional limitations [6].

Morquio syndrome, or mucopolysaccharidosis IV, primarily manifests with skeletal dysplasia. Affected individuals often exhibit features of short stature, kyphoscoliosis of the spine, flattening of the vertebrae, and irregular bone growth. Joint abnormalities are also common, with manifestations including hyperflexibility, hypermobility, laxity, and instability, which can contribute to impaired mobility and functional limitations [7]. Systemic involvement may also be present. Due to the grossly similar skeletal features observed between the two conditions, DMC is often referred to as pseudo-Morquio syndrome [8].

The overlapping clinical features between DMC (pseudo-Morquio syndrome) and Morquio syndrome pose diagnostic challenges, but the presence of progressive intellectual disability and the absence of corneal clouding and urinary GAGs help to rule out Morquio syndrome, which was our first consideration in this case. Additionally, the radiological evidence from X-rays of the wrist, spine, and pelvis, characteristically showing abnormal spine curvature and a lace-like pattern over the iliac crest, suggested a provisional diagnosis of DMC syndrome [8,9].

Genetic testing confirmed the diagnosis with a homozygous mutation in the *DYM* gene, which was pathogenic. This mutation is associated with DMC disease and a variant known as Smith-McCort dysplasia. Smith-McCort dysplasia is not associated with any intellectual impairment [10]. Management of these conditions typically involves a multidisciplinary approach aimed at addressing the specific symptoms and complications associated with skeletal dysplasia, with interventions ranging from supportive therapies to surgical procedures aimed at correcting skeletal deformities and improving overall quality of life [11,12].

## Conclusions

This study emphasizes the significance of a precise and definitive diagnosis between two such close possibilities with nearly indistinguishable clinical features. In skeletal dysplasias, a good clinical history and examination, a skeletal survey, and genetic testing are needed. In our case, confirmed genetic testing helped diagnose DMC syndrome. Accurate diagnosis is helpful in prenatal genetic testing.
